# Cerebral Organoids and Antisense Oligonucleotide Therapeutics: Challenges and Opportunities

**DOI:** 10.3389/fnmol.2022.941528

**Published:** 2022-06-27

**Authors:** Jenny Lange, Haiyan Zhou, Amy McTague

**Affiliations:** ^1^Department for Developmental Neurosciences, Zayed Centre for Research Into Rare Disease in Children, Great Ormond Street Institute of Child Health, University College London, London, United Kingdom; ^2^Genetics and Genomic Medicine Research and Teaching Department, Great Ormond Street Institute of Child Health, University College London, London, United Kingdom; ^3^NIHR Great Ormond Street Hospital Biomedical Research Centre, London, United Kingdom

**Keywords:** organoid, cerebral organoid, neurological disease, antisense oligonucleotide (ASO), RNA therapeutics

## Abstract

The advent of stem cell-derived cerebral organoids has already advanced our understanding of disease mechanisms in neurological diseases. Despite this, many remain without effective treatments, resulting in significant personal and societal health burden. Antisense oligonucleotides (ASOs) are one of the most widely used approaches for targeting RNA and modifying gene expression, with significant advancements in clinical trials for epilepsy, neuromuscular disorders and other neurological conditions. ASOs have further potential to address the unmet need in other neurological diseases for novel therapies which directly target the causative genes, allowing precision treatment. Induced pluripotent stem cell (iPSC) derived cerebral organoids represent an ideal platform in which to evaluate novel ASO therapies. In patient-derived organoids, disease-causing mutations can be studied in the native genetic milieu, opening the door to test personalized ASO therapies and n-of-1 approaches. In addition, CRISPR-Cas9 can be used to generate isogenic iPSCs to assess the effects of ASOs, by either creating disease-specific mutations or correcting available disease iPSC lines. Currently, ASO therapies face a number of challenges to wider translation, including insufficient uptake by distinct and preferential cell types in central nervous system and inability to cross the blood brain barrier necessitating intrathecal administration. Cerebral organoids provide a practical model to address and improve these limitations. In this review we will address the current use of organoids to test ASO therapies, opportunities for future applications and challenges including those inherent to cerebral organoids, issues with organoid transfection and choice of appropriate read-outs.

## Introduction

In recent decades significant advances have been made in the field of stem cell research. *In vitro* differentiation of iPSCs has allowed the generation of a vast array of cellular subtypes, recapitulating neuronal tissues which have until now been inaccessible (Takahashi and Yamanaka, 2006; Avior et al., 2016; Doss and Sachinidis, 2019). The use of patient-derived stem cells carrying pathogenic genetic variants has led to mechanistic insights in conditions including epilepsy, Parkinson disease and Alzheimer disease (Tidball and Parent, 2016; Simkin and Kiskinis, 2018; Caiazza et al., 2020; Penney et al., 2020). Prolonged periods of culture in traditional 2D *in vitro* environments have still failed to recapitulate stages of cellular maturity associated with late onset diseases (Ho et al., 2016; Gordon et al., 2021), thus limiting the extent to which disease can be modeled *in vitro*. Cells grown in 2D also fail to reproduce the 3D nature of *in vivo* environments and lack crucial cell-cell as well as cell-matrix interactions, limiting their usefulness (Doss and Sachinidis, 2019; de Leeuw et al., 2021). This has been a particular problem for modeling of neurological diseases, where neuronal subtypes and glial cells operate in complex cellular and functional networks.

The generation of stem cell derived mini organs and consequent advent of organoid research has revolutionized the way neurological disease is studied (Mariani et al., 2012; Lancaster et al., 2013, 2017; Pasca et al., 2015). Through application of developmental factors, as well as in some cases through the addition of extracellular matrices such as Matrigel, stem cells can self-organize or, through addition of small molecules, undergo directed differentiation into structures resembling that of the developing brain, replicating some of the cellular diversity observed in the central nervous system (Lancaster et al., 2013; Lancaster and Knoblich, 2014; Pasca et al., 2015; Trujillo and Muotri, 2018; Benito-Kwiecinski and Lancaster, 2020). Organoids therefore represent a major advancement to the field of neuroscience drug discovery, allowing testing of novel therapeutics and investigation of potential toxicity in a humanized, 3D model of specific brain regions (Costamagna et al., 2021).

Neurological disorders carry a significant health burden for individuals and society, and many, particularly childhood-onset disorders, remain without effective treatments despite decades of drug development using conventional models (Deuschl et al., 2020). Recent advances in the genetic underpinning of neurological disorders has led to precision therapeutic approaches, where the treatment is directly targeted to the etiology of the disorder (Gibbs et al., 2018). A number of gene-specific treatments including RNA-targeting therapies are under evaluation in clinical trials, such as for Dravet syndrome (Han et al., 2020; Laux et al., 2020) and are an approved treatment in spinal muscular atrophy (Passini et al., 2011; Hill and Meisler, 2021; Osredkar et al., 2021). However clinical trials of other ASO therapies for conditions such as Huntington’s disease have not been successful, despite promising pre-clinical studies (Tabrizi et al., 2019; Kwon, 2021; Rook and Southwell, 2022). Here we review the potential and challenges of using antisense oligonucleotides in brain organoid systems to better target neurological diseases.

## Antisense Oligonucleotides to Date—Design and Clinical Application

Antisense oligonucleotides (ASOs) are one of the most widely used approaches for targeting RNA and modifying gene expression. ASOs are single stranded DNA analogs, usually 13–25 bases long, that are designed to hybridize with target genes and have high specificity to pathogenic targets, thus reducing adverse effects associated with off-target effects (Chery, 2016; Zheng et al., 2018; Watts et al., 2019). ASOs may regulate gene expression through one of several mechanisms: mRNA cleavage, steric blocking causing translational arrest or pre-mRNA splice switching (Bennett et al., 2017; Scoles et al., 2019). Gene silencing is largely achieved by mRNA degradation through ribonuclease (RNase) H1 recruitment by ASOs (Crooke, 1999; Wu et al., 2004). Following ASO binding to target mRNA, an RNA-ASO hybrid is formed which induces enzymatic degradation by RNase H thus reducing mRNA levels (Furdon et al., 1989). Alternatively, ASOs can be targeted to pre-mRNA and regulate pre-mRNA splicing by binding to splice motifs, or exonic and intronic splicing enhancers (ESEs/ISEs) or exonic and intronic silencers (ESSs/ISSs) to lead to either exon-skipping or exon-inclusion (Siva et al., 2014; van der Wal et al., 2017). An exon-inclusion ASO approach has been successfully developed for Spinal Muscular Atrophy (SMA) (Finkel et al., 2017). Redirecting the pre-mRNA splicing complex to skip selected exons at an early transcriptional stage to obtain truncated protein has been a useful strategy in Duchenne Muscular Dystrophy where residual protein activity is sufficient to ameliorate disease progression (Charleston et al., 2018; Echevarría et al., 2018). An additional strategy has been Targeted Augmentation of Nuclear Gene Output (TANGO), in which an ASO alters splicing to exclude a poison exon which usually results in a non-productive transcript (Han et al., 2020; Lim et al., 2020). Mutation-specific splice switching ASO has also been developed in clinic for ultra-rare and fatal neurodegenerative conditions as the launching of “n-of-1” patient-customized therapy (Kim et al., 2019c).

However, major challenges still remain for ASO treatment in neurological disorders, due to rapid degradation, low cellular uptake or undesirable uptake by cell types other than those more affected by disease, and the tight blood brain barrier which blocks the penetration of ASO to the brain (Bennett and Swayze, 2010; Bennett et al., 2017; Dowdy, 2017).

Significant progress has been made in oligonucleotide chemical modification in the last several decades (Scoles et al., 2019; Gagliardi and Ashizawa, 2021; Gupta et al., 2021). This has been the major technology trigger for the rapid development and clinical translation of ASOs (Bennett and Swayze, 2010; Bennett et al., 2017; Dowdy, 2017). ASOs can be modified through a variety of chemical alterations, which confer increased resistance to degradation by nucleases and improved affinity to the binding target (Rinaldi and Wood, 2018; Dhuri et al., 2020). Modifications can provide ASOs with varying properties that may be combined, or in other instances may not be compatible (Dowdy, 2017). The wide use of phosphate backbone modifications in first generation ASOs improved resistance to endonucleases and bioavailability, and also increased binding affinity to the target RNA (Furdon et al., 1989). One of the first modifications was the inclusion of a hydrophobic phosphorothioate (PS), which increases circulation time due to increased resistance against nucleases in tissues and promotes binding affinity (Eckstein, 2000, 2014; Geary et al., 2015; Crooke et al., 2017). Several other backbone modifications have since been successfully integrated in next generation ASO designs (Duffy et al., 2020). Further modifications to sugar ribose (2′O-methyl and 2′O-methoxyethyl) have shown reduced toxicity whilst improving binding to the target RNA (Prakash, 2011). Highly modified ASOs such as morpholino oligomers, peptide nucleic acids (PNA) and locked nucleic acids (LNA) differ significantly from original oligonucleotide designs, and have also shown significant improvements in binding affinity, decreased susceptibility to degradation and improved diffusion (Summerton and Weller, 1997; Summerton et al., 1997; Pellestor and Paulasova, 2004; Hagedorn et al., 2018). Therefore extensive modifications to ASOs have been developed with distinct advantages for certain tissues, delivery strategies and diseases.

Various delivery technologies have been developed to promote the delivery and uptake of ASOs in different organs. Nucleic acid carrier systems, also known as nano carriers, have been more widely used for both siRNA and ASOs (Zhu et al., 2015; Ramasamy et al., 2021). These include polymeric-nanoparticle based systems which can increase their delivery potential through decreasing endosomal entrapment of ASOs (Danhier et al., 2012; Cai et al., 2017). However increased toxicity and non-specific interactions have hindered the advancements of this technology (Dhuri et al., 2020). Lipid-based systems, such as lipid nanoparticles have been successfully used to deliver ASOs (Rehman et al., 2013; Kumar et al., 2014). As these are typically coated with polyethylene glycol, blood circulation time of the ASO-lipid nanoparticle complex is increased. Lastly a major limitation of current ASO technology is that ASOs do not readily pass the blood brain barrier with current options limited to intrathecal or intravitreal administration for retinal diseases (Bochot et al., 2000; Mazur et al., 2019). However a recent study delivered ASOs across the mouse blood brain barrier using a glucose-coated polymeric nanocarrier (Min et al., 2020).

In recent years, several ASO drugs have been approved for clinical use in neurodegenerative conditions, such as for spinal muscular atrophy, familial amyloid polyneuropathy and Duchenne muscular dystrophy (Table 1). Despite these notable successes, in several instances clinical trials had to be terminated due to safety concerns or lack of drug efficacy. The Phase III study of tominersen for Huntington disease was stopped prematurely in 2021 following promising pre-clinical and Phase I/II data although it may be relaunched (Kwon, 2021). Phase I/II trials of two other HD ASOs were also paused shortly afterward. The use of disease-relevant patient-derived modeling systems may be an important factor in the initial screening of ASO efficacy and toxicity, and could be an advantage for screening allele-specific ASOs. iPSC-based *in vitro* systems have yielded important results, however, their two dimensional nature and often single cell type, fails to replicate the complex architecture of the human brain (Nizzardo et al., 2015; Lee and Huang, 2017; Mathkar et al., 2019; Hauser et al., 2022). Brain organoids, particularly those which are patient-derived, could therefore be a crucial model for investigating biodistribution, toxicity and efficacy of ASOs in a more disease relevant environment.

**TABLE 1:** Currently approved oligonucleotide therapies.

Disease	Drug (market) names	FDA^+^/EMA^#^ approved	Administration	Mechanism	Chemistry
Acute hepatic porphyria	Givosiran (Givlaari)	2019^+^	Subcutaneous injection	RNA interference	21/23 mer Dicer substrate siRNA
CLN7 disease (Batten disease)	Milasen	2018^+^	Intrathecal injection	Splice modulation	22 mer Phosphorothioate 2′-*O*-methoxyethyl
Cytomegalovirus retinitis *withdrawn from EU & US	Fomivirsen (Vitravene)	1998	Intravitreal injection	Blocks translation of mRNA	21 mer Phosphorothioate DNA
Duchenne muscular dystrophy	Eteplirsen (Exondys 51)	2016^+^	IV infusion	Exon skipping (Splice modulation)	30 mer Phosphorodiamidate morpholino oligomer
	Golodirsen (Vyondys 53)	2016^+^	IV infusion		25 mer Phosphorodiamidate morpholino oligomer
	Viltolarsen (viltepso)	2020^+^	IV infusion		21 mer Phosphorodiamidate morpholino oligomer
	Casimersen (Amondys 45)	2021^+^	IV infusion		22 mer Phosphorodiamidate morpholino oligomer
Familial chylomicronemia syndrome	Volanesorsen (Waylivra)	2019^#^	Subcutaneous injection	RNase H degradation	20 mer 2′-*O*-methoxyethyl
Hereditary transthyretin-mediated amyloidosis	Inotersen (tegsedi)	2018^+^	Subcutaneous injection	RNase H degradation	20 mer Phosphorothioate 2′-*O*-methoxyethyl
	Patisiran (Onpattro)		IV infusion	RNA interference	19 + 2 mer 2′-*O*-methyl
Homozygous familial hypercholesterolemia	Mipomersen (Kynamro)	2013^+^	Subcutaneous injection	RNase H degradation	20 mer phosphorothioate 2′-*O*-methoxyethyl
Neovascular age related macular degeneration	Pegaptanib (Macugen)	2004^+^	Intravitreal injection	Binds and blocks receptors	27 mer 2′-F/2′-*O*-methyl pegylated
Spinal muscular atrophy	Nusinersen (Spinraza)	2016^+^	Intrathecal injection	Exon skipping (Splice modulation)	18 mer Phosphorothioate 2′-*O*-methoxyethyl
Veno-occlusive disease in liver	Defibrotide (Defitelio)	2016^+^	IV infusion	Modulates function of cationic proteins	Phosphodiester ssDNA and dsDNA

*^+^Approved by FDA and EMA approved. ^#^EMA approved only.*

## Advances in Organoid Generation

**FIGURE 1:**
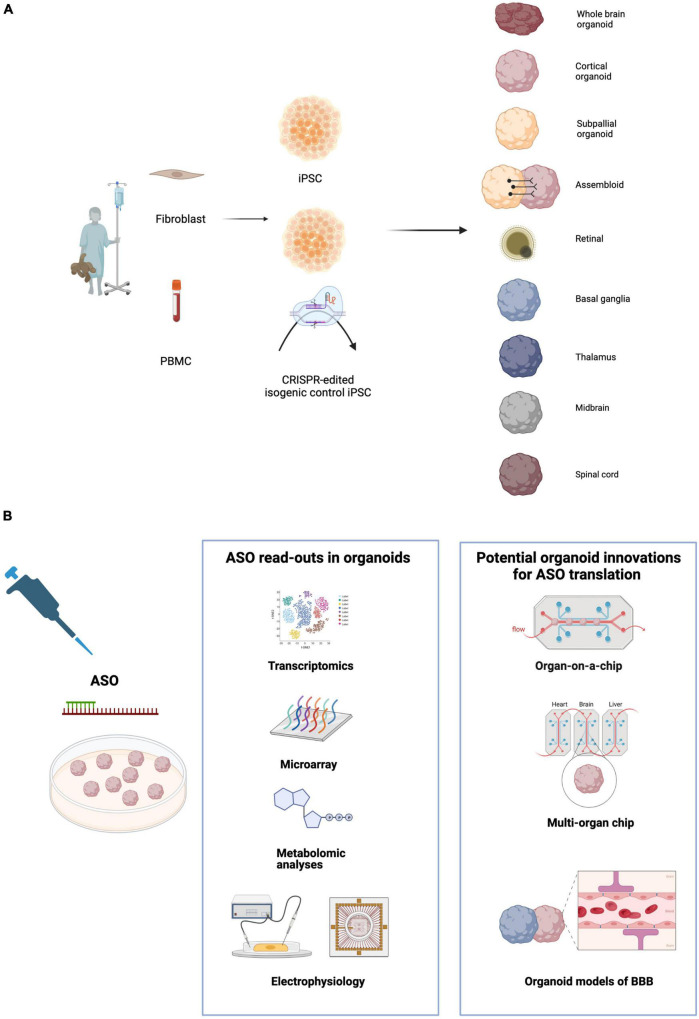
**(A)** Induced pluripotent stem cell (iPSC) derived cerebral organoids model numerous brain regions and cell types. Patient-derived cells including fibroblasts and PNMCs (peripheral blood mononuclear cells) are reprogrammed to iPSCs. CRISPR-Cas9 editing generates isogenic controls where the pathogenic variant of interest is corrected to wild-type. iPSCs can be differentiated to a range of organoids representing diverse regions of the central nervous system. Assembloids are fusions of organoids which combine multiple brain regions to model interactions and circuit formation. **(B)** Organoids offer a platform for multiple readouts of ASO efficiency and toxicity. Transcriptomic analysis using microarray, RNA-seq or single cell RNA-seq can assess ASO efficacy and off target effects. Metabolomic testing and electrophysiology are important assays of phenotypic rescue by ASOs. Microfluidic technology can be used to generate organoids on a chip and multi-organ chips allowing control of the organoid micro-environment, increasing reproducibility and enabling testing of efficacy and toxicity in multiple cell types. Modeling of BBB (blood brain barrier) using vascularized organoids will also be an important step toward ASO translation.

Human iPSC derived three-dimensional (3D) organoids have significantly advanced our ability to model neurodevelopment of the human brain (Lancaster et al., 2013, 2017; Pasca et al., 2015). Various studies have shown the ability to drive organoids toward specific brain regions, including cerebellar, hippocampal, midbrain, striatal and spinal cord organoids (Muguruma et al., 2015; Sakaguchi et al., 2015; Jo et al., 2016; Qian et al., 2016; Bagley et al., 2017; Khong et al., 2019; Kim et al., 2019d; Miura et al., 2020; Figure 1A). These have shown complex self-organization of cytoarchitectural structures that resemble the developing brain (Tian et al., 2020; Bhattacharya et al., 2022). This is of particular advantage when studying developmental disorders or the effect of viruses such as ZIKA virus that affect structure and function of the developing central nervous system (Lancaster et al., 2013; Qian et al., 2016; Trujillo and Muotri, 2018; Xu et al., 2021). Unlike rodent brains, organoids not only recapture the temporal sequence of cortical development but also diversity of neural progenitor cells of the human developing brain (Florio and Huttner, 2014; Toma et al., 2016; Sidhaye and Knoblich, 2021), thus providing a better model for diseases affected by progenitor cell dysfunction such as microcephalies.

Fusion of organoids (assembloids) directed toward different brain regions, for example dorsal and ventral telencephalic organoids, have been successfully used to study migration of cortical interneurons, the formation of neural circuits and disruption thereof in disease (Bagley et al., 2017; Birey et al., 2017). For example, using cortico-striatal assembloids to model Phelan-McDermid syndrome, Miura et al. (2020) identified circuit abnormalities which were not observed in striatal organoids alone. Whilst organoids to date have largely been used to study neurodevelopmental diseases (Trujillo and Muotri, 2018; Setia and Muotri, 2019), they have provided some insight into neurodegenerative disease (Wray, 2021). Organoids can be maintained in culture for prolonged periods compared to 2D neuronal cultures, allowing emergence of mature cell types and improving modeling of cellular interactions. 3D models of tauopathies (Choi et al., 2014; Bowles et al., 2021), Parkinson’s disease (Kim et al., 2019a), Huntington’s disease (Conforti et al., 2018) and Creutzfeld-Jacobs disease (Groveman et al., 2019, 2021) have successfully delineated novel disease phenotypes and replicated pathology observed in murine and *in vitro* models (reviewed in Barral and Kurian, 2016; Paşca, 2018).

## Testing of Antisense Oligonucleotides in Organoid Models to Date

Organoids provide an innovative platform for drug discovery in neurological disease and have successfully been used to test small molecules and repurposed drugs for microcephalies, Zika virus (Dang et al., 2016; Watanabe et al., 2017), schizophrenia (Stachowiak et al., 2017), Alzheimer’s disease (Choi et al., 2014; Zhao et al., 2020; Alić et al., 2021), Frontotemporal Dementia (FTD) (Szebényi et al., 2021) and Creutzfeldt-Jacobs disease (Groveman et al., 2021), albeit none have yet been translated to clinical trials.

A small number of studies have thus far used organoids to investigate the effect of ASOs on disease phenotypes. Organoid tumor models have been targeted with ASOs with promising results. Mammary tumor organoids were treated with ASOs targeting transcripts of Mammary Tumor Associated RNAs (MaTARs) achieving 30–68% knockdown (Diermeier et al., 2016). MaTARs were defined by the authors as a subgroup of long non-coding RNAs that were overexpressed in mammary tumor sections, compared to healthy tissue, as determined by RNA sequencing. Several ASOs reduced branching morphogenesis and morphological defects in tumor organoids (Diermeier et al., 2016). In murine and patient-derived gastric tumor organoids, knockdown of microRNA130b, which was shown to be involved in the T-cell suppressor phenotype and metaplasia, resulted in reduced tumor volume (Ding et al., 2020).

To date, only one study has investigated ASOs in a cerebral organoid model. Using *MAPT* mutant iPSC lines derived from frontotemporal dementia (FTD) patients and corresponding isogenic controls, telencephalic organoids were generated which replicated the selective loss of glutamatergic neurons and early dysfunction of the autophagy-lysosomal pathway observed in 2D neuronal cultures and *in vivo* (Bowles et al., 2021). Mutant lines showed increased susceptibility to glutamate toxicity, which was not apparent in isogenic controls. PIKFYVE (Phosphoinositide Kinase, FYVE-Type Zinc Finger Containing), a lipid kinase that regulates endo-lysosomal trafficking, was targeted with ASOs to suppress gene expression in order to protect against glutamate toxicity. Organoids were exposed to 10 μM ASOs for 48 h. Expression of PIKFYVE was significantly reduced in organoids with increased survival of glutamate-treated neurons. This effect was only detected in patient organoids and not seen in isogenic controls. Although limited to one study, this is a promising start for the use of cerebral organoids to investigate novel ASO strategies.

Compared to cerebral organoid, retinal organoids have been more widely used as a relevant test system of ASO therapeutics. Patient iPSC-derived retinal organoids allow the direct study of mutation-specific ASO complementary to the human gene. This is particularly important when targeting mutations leading to altered splicing, as splicing mutations in human gene may not be recognized by the splicing machinery of other species. It has previously been observed that a human knock-in transgenic mouse containing the CEP290 c.2991+1655A>G allele is processed differently by the mouse photoreceptors with unexpected splicing isoforms created as the mouse photoreceptors have different splicing machinery (Garanto et al., 2013). In addition, human retinal organoids allow retina-specific off-target effects to be studied and they also allow an estimation of the necessary effective intravitreal concentration of therapeutic, which can be used to estimate clinical doses in human clinical trials (Dulla et al., 2018).

In retinal organoids, ASOs have been used to either correct splicing defects incurred by deep-intronic variants or to create truncated proteins with residual functions. Several ASOs have been developed for *CEP290*-associated Leber congenital amaurosis, restoring gene expression through altered pre-mRNA splicing which was confirmed in both animal models and iPSC-derived retinal organoids where 10 μM morpholino ASO where delivered directly to cells in the media (Parfitt et al., 2016; Kruczek and Swaroop, 2020). ASOs not only restored gene expression but also ciliogenesis without any off-target effects and clinical trials are ongoing with promising results (Xue and MacLaren, 2020). ASO mediated exon skipping has proven successful in producing a shortened version of the protein usherin in iPSC-derived retinal organoids modeling retinitis pigmentosa, with 10 μM phosphorodiamidate morpholino oligonucleotide delivered in cell media (van Diepen et al., 2019; Dulla et al., 2021). A clinical trial is now underway. Finally, splicing defects incurred in Stargardt disease, a progressive retinal disorder, could be rescued in 3D retinal organoids by 1 μM ASO administration targeted the affected gene *ABCA4* (Khan et al., 2020). ASOs for *ABCA4* were designed with a phosphorothioate backbone and a 2′O-methyl sugar modification. Retinal organoids have proved an excellent model for the testing of novel therapies and most importantly ASOs. The success can partially be attributed to the highly penetrant, monogenic nature of these retinal diseases and the somewhat less cellularly diverse nature of retinal organoids compared to cerebral organoids (Kruczek and Swaroop, 2020). Nevertheless, it highlights the great potential of organoids as disease and drug testing models.

## Future Directions and Challenges

### Organoid Reproducibility

Although great strides have been taken in organoid research, there remains a variety of challenges for ASO testing in organoids. Organoids can be costly and labor-intensive to generate and difficult to scale up for higher throughput assays which would be attractive to pharma companies (Costamagna et al., 2021). Recently, Narazaki et al. (2022) have addressed this by using biocompatible polymers that prevent organoid-organoid fusion, successfully screening 298 drugs in over 2,400 cortical organoids. Whilst this is one step in the right direction to make organoids more attractive to large scale studies, the inherent cellular heterogeneity poses significant technical challenges (Costamagna et al., 2021). Sources of variability in organoids that may confound the outcomes of ASO treatment on a larger scale include the genetic background of iPSC lines, with genomic instability induced by cell passaging as well as batch to batch variations in differentiation efficiency (Rouhani et al., 2014; Burrows et al., 2016; Kyttälä et al., 2016; Pollen et al., 2019; Bhaduri et al., 2020; Brancati et al., 2020). Additionally, a high degree of heterogeneity of cell types and organoid maturity may be exhibited within a single batch thus making reproducibility of results another issue.

### Representation of Cell Types

A further challenge for the testing of ASOs in cerebral organoids is the adequacy of representation of cell types and thus the neuronal micro-environment. Single cell RNA sequencing has also shown a limited range of gene expression in organoids compared to human brains, suggesting that the true complexity of the brain is not being captured (Bhaduri et al., 2020). This includes the adequate representation of glial cells in the brain where they thought to represent a 1:1 ratio with neurons (von Bartheld et al., 2016). Whilst astrocytes are found in the majority of organoids usually from 1 month of organoid differentiation (Pasca et al., 2015; Sloan et al., 2017; Bhattacharya et al., 2022), the presence of oligodendrocytes occurs much later after 3 months (Kim et al., 2019b). Despite modified protocols to encourage oligodendrocyte differentiation (Madhavan et al., 2018; Marton et al., 2019), further improvements are necessary for the development of robust, structured myelin. Microglia are typically absent from forebrain organoids as they are of mesodermal origin (Ginhoux et al., 2010) and require specialized protocols which have recently been developed (Xu et al., 2021). The role of glial cell types in neurological disease is emerging, highlighting the importance of organoids encompassing all cell types so that all affected cell types can be ASO treated (Zuchero and Barres, 2015; Bernaus et al., 2020; Kim et al., 2020). Although data is limited, there is evidence in mice that ASO potency may differ between brain regions (Nikan et al., 2020) or even neurons and glia (Jafar-Nejad et al., 2021). This could have a significant effect on the outcome of ASO treatment depending on the disease and cell-type specific vulnerabilities. However the ability to direct cerebral organoid differentiation into a particular cell type also represents a strength of this system, as it would allow assessment of phenotypic rescue in organoids of differing regional identities as well as fused assembloids (Figure 1A).

### Organoids and the Neurovascular System

Further challenges include the neurovascular system in organoids, as endothelial cells and pericytes are not usually found in organoids due to their non-neural origin (Brancati et al., 2020). The neurovascular system has crucial homeostatic functions including oxygen supply. The absence thereof leads to internal necrosis in organoids due to reduced oxygen diffusion and inadequate nutrient delivery, as well as waste accumulation (Lancaster et al., 2013; Del Dosso et al., 2020; Matsui et al., 2021; Bhattacharya et al., 2022). Of course, adequate diffusion through organoids is also of major importance for successful ASO delivery. The use of spinning bioreactors has been shown to improve nutrient diffusion (Qian et al., 2018). Several studies have made efforts to vascularize organoids, for example to introduce endothelial differentiation, whilst other have exogenously added endothelial cells, mesodermal progenitors and pericytes to brain organoids (AlFatah Mansour et al., 2018; Bergmann et al., 2018; Nzou et al., 2018; Cakir et al., 2019; Shi et al., 2020; Matsui et al., 2021). However none of these models replicated flow and perfusion of the natural structure; this is of particular importance when studying distribution of ASOs. Recent studies have generated organoids that included formation of the choroid plexus (ChP), which is crucial to brain developmental and secreted cerebrospinal fluid (Pellegrini et al., 2020). It forms the blood-CSF barrier, a structure similar to the BBB, preventing toxic substances from reaching the brain. These organoids form tight barriers with similar permeability to the brain, when several compounds were tested. Given these recent advances, organoids may soon offer a platform in which to test the ability of ASOs to traverse the BBB in disease states (Figure 1B).

BBB spheroids have been used shown to accurately recapitulate BBB function and have been used to screen for brain penetrant peptides (Cho et al., 2017). Organoids accurately modeling the blood brain barrier lend themselves to testing molecules that facilitate blood brain barrier opening such as angubindin-1, which has been shown to safely allow for ASO delivery in the mouse brain (Zeniya et al., 2018). Similarly, ASO delivery by tumor cell derived small apoptotic bodies has shown high delivery efficiency in mouse brains (Wang et al., 2021) and organoids could provide a valuable human model to test safety and efficacy of this method. Endogenous peptides such as Neurotensin, have also been shown to improve cellular uptake and activity of ASOs (Nikan et al., 2020). The concept of an “organ on a chip” or 3D printing of organs, combining microfluidic systems and specialized extracellular matrices, has generated great interest and would meet the challenge of replicating CSF flow in models of the blood brain barrier (Figure 1B). This could be highly relevant for toxicity testing using patient-derived organoids and may allow the recreation of the brain “physiome” (Seo et al., 2022).

### Personalized Therapies

A great strength of patient-derived iPSC models is that they pave the way for personalized therapies, such as recent “n of 1 studies” including the generation of a personalized antisense therapy for a severe form of neuronal ceroid lipofuscinosis (Kim et al., 2019c). A patient-derived organoid system could allow both testing of these individualized therapies and assessment of off-target and toxicity effects, potentially offering a rapid route to translation for life-threatening disorders. There is an emerging school of thought that some of the challenges encountered in non-specific gene knockdown such as in the Huntingtons disease study may be improved by allele-specific approaches (Pfister et al., 2009; Carroll et al., 2011; Østergaard et al., 2013). A patient-derived organoid model would allow investigation of allele-specific ASOs, for example by targeting SNPs present on the mutant but not the wild-type allele.

## Conclusion

While cerebral organoids offer many opportunities for ASO testing, they possess inherent limitations. Cerebral organoids have been shown to usefully model neurodevelopmental processes, but as with other iPSC-derived neuronal models, they possess limitations for later-onset or neurodegenerative conditions. Although assembloids allow for modeling of neuronal circuits and spontaneous network activity has been observed in several organoid models (Trujillo et al., 2019; Zourray et al., 2022), sufficient levels of maturity are not reached to fully reflect network activity in the human brain. However, it is clear that advances in organoid differentiation are continuing at pace with improvements in patterning, reproducibility and vascularization. As a complement to 2D and animal models, they offer an exciting opportunity to bring personalized, precision RNA therapies for neurological disorders from the lab to the clinic.

## Author Contributions

JL, HZ, and AM contributed to the conception, preparation of the first draft, and manuscript revision. All authors contributed to the article and approved the submitted version.

## Author Disclaimer

The views expressed are those of the authors and not necessarily those of the NHS, the NIHR or the Department of Health. Figures were created with BioRender.com. For the purpose of open access, the author has applied a Creative Commons Attribution (CC BY) license to any author accepted manuscript version arising.

## Conflict of Interest

AM has provided consultancy *via* UCL Consultants for Biogen. The remaining authors declare that the research was conducted in the absence of any commercial or financial relationships that could be construed as a potential conflict of interest.

## Publisher’s Note

All claims expressed in this article are solely those of the authors and do not necessarily represent those of their affiliated organizations, or those of the publisher, the editors and the reviewers. Any product that may be evaluated in this article, or claim that may be made by its manufacturer, is not guaranteed or endorsed by the publisher.
